# Genome-Wide Linkage Scan for Primary Open Angle Glaucoma: Influences of Ancestry and Age at Diagnosis

**DOI:** 10.1371/journal.pone.0021967

**Published:** 2011-07-12

**Authors:** Kristy R. Crooks, R. Rand Allingham, Xuejun Qin, Yutao Liu, Jason R. Gibson, Cecilia Santiago-Turla, Karen R. Larocque-Abramson, Elizabeth Del Bono, Pratap Challa, Leon W. Herndon, Stephen Akafo, Janey L. Wiggs, Silke Schmidt, Michael A. Hauser

**Affiliations:** 1 Center for Human Genetics, Duke University Medical Center, Durham, North Carolina, United States of America; 2 Department of Ophthalmology, Duke University Eye Center, Duke University Medical Center, Durham, North Carolina, United States of America; 3 Department of Ophthalmology, Harvard Medical School and Massachusetts Eye and Ear Infirmary, Boston, Massachusetts, United States of America; 4 Unit of Ophthalmology, Department of Surgery, University of Ghana Medical School, Accra, Ghana; Ohio State University Medical Center, United States of America

## Abstract

Primary open-angle glaucoma (POAG) is the most common form of glaucoma and one of the leading causes of vision loss worldwide. The genetic etiology of POAG is complex and poorly understood. The purpose of this work is to identify genomic regions of interest linked to POAG. This study is the largest genetic linkage study of POAG performed to date: genomic DNA samples from 786 subjects (538 Caucasian ancestry, 248 African ancestry) were genotyped using either the Illumina GoldenGate Linkage 4 Panel or the Illumina Infinium Human Linkage-12 Panel. A total of 5233 SNPs was analyzed in 134 multiplex POAG families (89 Caucasian ancestry, 45 African ancestry). Parametric and non-parametric linkage analyses were performed on the overall dataset and within race-specific datasets (Caucasian ancestry and African ancestry). Ordered subset analysis was used to stratify the data on the basis of age of glaucoma diagnosis. Novel linkage regions were identified on chromosomes 1 and 20, and two previously described loci—GLC1D on chromosome 8 and GLC1I on chromosome 15—were replicated. These data will prove valuable in the context of interpreting results from genome-wide association studies for POAG.

## Introduction

Glaucoma comprises a group of disorders that are characterized by retinal ganglion cell death and a characteristic pattern of progressive vision loss. POAG is the most common type of glaucoma globally [1], and it is estimated that by 2020 the number of people diagnosed with POAG in the United States alone will total more than 3 million [2]. It has long been recognized that there is a heterogeneous genetic component to POAG. Genome-wide linkage analyses have identified 14 loci, designated GLC1A-N, which are thought to contribute to POAG risk [2]–[18]. Causative mutations have been identified in genes within three of these loci: Myocilin (MYOC) on 1q24.3 (GLC1A) [12], optineurin (OPTN) on 10p15-14 (GLC1E) [8], and WD40-repeat 36 on 5q22.1 (GLC1G) [6]. Together, mutations in these three genes account for less than 10% of POAG cases [19]. Thus, the majority of the genetic etiology of POAG remains to be discovered.

Among the challenges in the study of POAG is genetic and phenotypic heterogeneity of study subjects. For example, while mutations in OPTN or MYOC both result in POAG, a missense change in OPTN (E50K) causes an adult-onset form of POAG that is characterized by normal intraocular pressures [8], whereas MYOC mutations can cause either adult-onset or juvenile-onset disease with highly elevated intraocular pressures [20]. Reducing this genetic variability in the study population is essential for identifying variants that contribute to POAG risk, and it can be achieved by phenotypic stratification. In the study of POAG and other complex diseases, phenotypic stratification by ordered subset analysis (OSA) [21] has been particularly successful in identifying genetically homogeneous subsets of families with increased evidence for linkage and in reducing linkage intervals for follow-up analysis. In OSA, families are ranked according to a phenotypic variable. In this study, families were sorted from lowest to highest average age at diagnosis (AAD, see Methods) of POAG in affected relatives. We chose this variable based on previous linkage analyses by our group [3] and others [22] that established AAD as an important source of genetic heterogeneity.

In this study we report the results of the largest SNP-based genome-wide POAG linkage study performed to date. Using both standard linkage methodology and OSA to account for genetic heterogeneity, our study identified global as well as ancestry-specific and phenotype-specific genomic regions that may harbor POAG susceptibility variants.

## Results

### Clinical data summary

Table 1 summarizes sample size and clinical characteristics of the study population. After exclusion of families segregating MYOC mutations, 786 sampled subjects from 134 multiplex families were analyzed. Clinical characteristics were similar among subjects of African ancestry and Caucasian ancestry. As expected, intraocular pressure (IOP) was clinically elevated in affected members of both ancestry groups, and pressures in those of African ancestry were significantly higher than those of Caucasian ancestry (31.5±1.0 versus 28.0±0.4 mmHg, p≤0.003). Both groups had an age at diagnosis (AAD, see Methods) that averaged in the 50 s and ranged from the 20 s to the 80 s. Slightly more than half the affected study subjects were female in both groups.

**Table 1:** Clinical characteristics of study populations.

		Caucasian ancestry	African ancestry	Total
**Families**	N	89	45	134
**Individuals**	N	538	248	786
**Affected**	N	278	123	401
	Mean age-of-onset in years (range)	59 (28–84)	54 (26–81)	58 (26–84)
	% female	54	56	54
	Mean maximum IOP	28.1	31.5	29.0
	Affected sibling pairs (N)	180	68	248
	Affected non-sib relative pairs (N)*	58	35	93
**Unaffected**	N	260	125	385
	Mean age-at-exam in years (range)	68 (50–83)	64 (45–76)	67 (45–83)
	% female	51	49	50

### Whole genome linkage analysis

**Figure 1:**
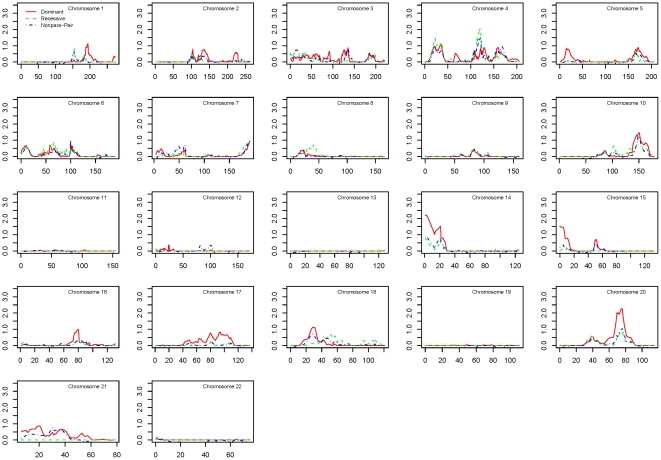
Linkage results for the combined dataset. Multipoint linkage scores for parametric dominant (red), parametric recessive (green), and nonparametric (blue) models are plotted for the combined dataset. Symbols indicate two-point lod scores ≥1.5 in the parametric dominant (red) and recessive (green) models.

Figure 1 shows results of the multipoint linkage analyses for the overall dataset, based on 5,233 SNPs with an average intermarker distance of 0.68 cM. Parametric linkage analyses were performed using both dominant and recessive models. We found the strongest evidence for linkage at 20q13.12–13.13, with the peak marker rs911411 (multipoint HLOD = 2.3, 75.8 cM, dominant model). The one-lod unit support interval comprises the region between the markers rs765147 and rs718630.

**Figure 2:**
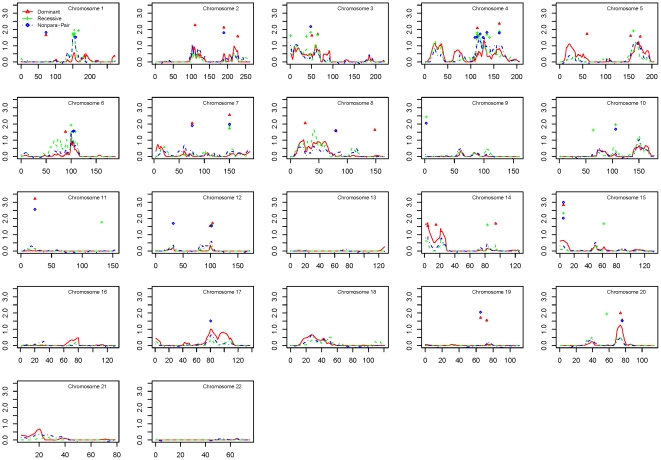
Linkage results for the Caucasian ancestry dataset. Multipoint linkage scores for parametric dominant (red), parametric recessive (green), and nonparametric (blue) models are plotted for the Caucasian ancestry dataset. Symbols indicate two-point lod scores ≥1.5 in the parametric dominant (red) and recessive (green) models.

**Figure 3:**
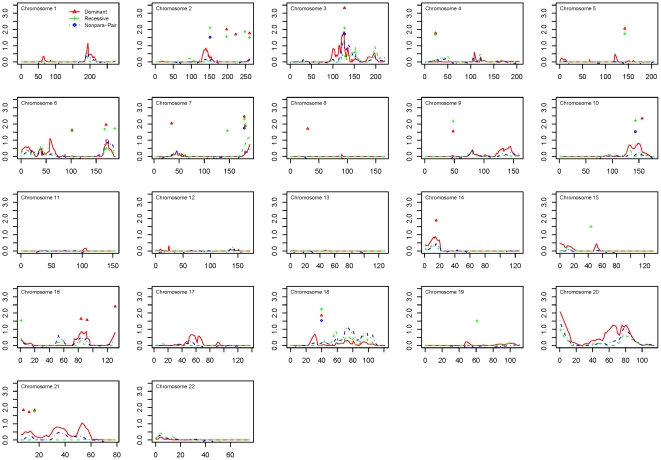
Linkage results for the African ancestry dataset. Multipoint linkage scores for parametric dominant (red), parametric recessive (green), and nonparametric (blue) models are plotted for the African ancestry dataset. Symbols indicate two-point lod scores ≥1.5 in the parametric dominant (red) and recessive (green) models.

Results of the two-point and multipoint linkage analyses for the Caucasian ancestry dataset and African ancestry dataset are shown in Figures 2 and 3, respectively. Among families of Caucasian ancestry, the best evidence for linkage was identified at 1q22–23.3, with the peak marker rs876537 (multipoint HLOD = 2.03, 154.5 cM, recessive model), and the one-lod unit support interval between markers rs2066981 and rs836. Among families of African ancestry, the strongest linkage was found at 20ptel-13, with the peak marker rs1342137 (multipoint HLOD = 2.09, 0.3 cM, dominant model), and the centromeric one-lod unit support boundary at rs600832.

The most notable linked regions for each dataset are shown in Table 2. In the combined dataset, there were four regions with multipoint lod scores >1.5. For the ancestry-specific datasets, all linked regions with both a multipoint lod score >1.5 and at least one two-point lod >1.5 within the one-lod support interval are shown. There were four such regions of interest in the Caucasian ancestry dataset, and two in the African ancestry dataset.

**Table 2:** Linkage analysis results.

Dataset	Chromosome	Location (cM)	SNP	peak multipoint lod (model)	peak 2-point lod
Combined	20q13.13	67–78	rs911411	2.3 (dom)	N/A
	14q11.2	0–12	rs1959344	2.2 (dom)	N/A
	4q26	111–124	rs814397	2.0 (rec)	N/A
	15q11.2	0–14	rs1722791	1.5 (dom)	N/A
					
Caucasian ancestry	1q23.2	148–158	rs876537	2.0 (rec)	1.8
	4q25	111–126	rs701760	1.9 (rec)	1.8
	14q11.2	0–28	rs1713423	1.8 (dom)	1.6
	6q15	98–114	rs1979797	1.6 (rec)	1.9
					
African ancestry	20p13	0–10	rs1774116	2.1	1.5
	3q13.32	119–130	rs1348969	1.8 (dom)	3.3

For the combined dataset, all linked regions with a peak multipoint lod >1.5 are shown. For the ancestry-specific datasets, all linked regions with both a multipoint lod >1.5 and at least one two-point lod ≥1.5 within the one-lod support interval are shown.

### Ordered subset analysis

**Figure 4:**
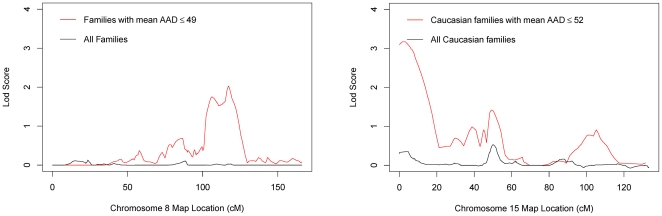
OSA results for chromosomes 8 and 15. Ordered subset analysis (red line) indicates improved evidence for linkage to chromosome 8 (left panel) among families with a mean age of onset ≤49 years. The black line plots non-parametric linkage analysis in all families in the combined dataset. Improved evidence for linkage to chromosome 15 (right panel) was demonstrated among Caucasian families with a mean age of onset ≤52 years. The black line plots non-parametric linkage analysis in all Caucasian families.

To examine whether AAD was a significant modifier of POAG linkage evidence, we performed OSA on the overall dataset, using ascending family mean AAD (lowest to highest age at diagnosis) as the covariate. We found significantly increased evidence for linkage to chromosomes 8 and 15 (empirical p≤0.05, with 10,000 permutations, Figure 4).

On chromosome 8 (Figure 4, left panel), the one-lod unit support interval comprised 22.1 cM with a maximum multipoint lod score of 2.03 at 117.2 cM (empirical p = 0.04). This region includes the previously reported POAG locus GLC1D [14]. All 26 families (13 Caucasian ancestry, 13 African ancestry) in the OSA subset had an average AAD below 50 years (mean 41, range: 28 to 49). In the complementary subset of 102 families (78 Caucasian ancestry, 34 African ancestry), the mean AAD was 62 years (range: 49 to 78).

The linkage region on chromosome 15 was found in the Caucasian early-onset dataset at 15q11–12 (Figure 4, right panel), comprising 13.6 cM with a maximum lod score of 3.17 at 3.2 cM (empirical p = 7×10^−5^), replicating our previously described GLC1I locus [3]. There were 19 families in this OSA subset, including the previously screened 11 families, all of which had an AAD below 53 years (mean: 45 years, range: 30 to 52 years). The complementary group of 68 families had an average age of onset of 63 years (range 52–78 years).

## Discussion

We have conducted family-based linkage analysis to identify regions of the genome that may harbor POAG susceptibility variants. These linkage regions provide distinct and complementary data that can assist in the interpretation of genome-wide association studies. The overall dataset and the African ancestry dataset linked POAG to non-overlapping intervals on chromosome 20. As these regions appear to be distinct from the JOAG-linked locus GLC1K, located between D20S846 and rs6081603 [23], there may now be as many as three discrete regions of interest on this chromosome alone. The Caucasian ancestry dataset linked POAG to a novel locus on chromosome 1.

Two of the regions with evidence for linkage, 14q11.2 in the combined and Caucasian ancestry datasets and 20p13 in the African ancestry dataset, are telomeric. It is generally accepted that telomeric regions may give rise to false positive linkage peaks at a higher frequency than other regions of the chromosome. In the absence of confirmation, these regions of interest should be considered with caution and follow-up analysis should be delayed until the findings are replicated.

Using OSA, we found increased evidence for linkage in families with adult early-onset on chromosomes 8 and 15, replicating previous findings. The linkage region on chromosome 8 includes the GLC1D locus, which was first reported in a single family with an apparently Mendelian form of glaucoma [14]. It is not surprising that the one-lod unit support interval calculated here is somewhat larger than the reported GLC1D locus, considering the differences between a nonparametric linkage analysis of a complex phenotype in a larger family collection, compared to recombination-based linkage mapping within a single large pedigree. Interestingly, the age-at-onset of glaucoma in that pedigree is reported as “within the third to fourth decade of life” which is consistent with the AAD of the OSA-identified subset of families reported here. Our finding suggests the possibility that one or more variants in the GLC1D region may give rise to both rare Mendelian and more common non-Mendelian forms of early-onset POAG.

We have previously reported microsatellite linkage to proximal 15q in a collection of 15 (11 Caucasian and 4 African American) early onset families [3]. Our current dataset comprises 34 early-onset families (19 Caucasian ancestry, 15 African ancestry). With the additional families, we were able to divide the dataset based on ancestry and analyze the two groups separately. We replicated linkage to GLC1I in the Caucasian dataset (19 families, including the 11 families reported earlier). In the current report, the one-lod unit support interval is larger and the peak lod score marginally lower than in the previous report. This likely reflects the nature of the individually more informative microsatellite markers compared to SNPs, rather than different underlying genes in the two OSA subsets. There was no evidence for linkage to POAG in the African ancestry early-onset dataset.

In conclusion, we have reported results of the first SNP-based genome-wide linkage analysis of POAG. We identified regions of interest for further investigation in a dataset of African ancestry, a dataset of Caucasian ancestry, and in the combined dataset. We also replicated two previously-reported early-onset POAG loci, strengthening the case that these regions may harbor one or more genes that are either causative for early-onset glaucoma or that modulate the age at which symptoms are first evident. We expect that the results reported here will prove useful in the context of interpreting and strengthening results from genome-wide association studies and will complement efforts to better understand the complex genetic etiology of glaucoma.

## Materials and Methods

### Subjects

This study adhered to the tenets of the Declaration of Helsinki. Written informed consent was obtained from all participating individuals. Caucasian and African-American subjects were recruited at the Duke Eye Center (Durham, NC). Caucasian subjects were also enrolled at the Massachusetts Eye and Ear Infirmary (Boston, MA). African subjects were enrolled at the University of Ghana. The research was reviewed and approved by the Institutional Review Board from all participating institutions, including Duke University Medical Center, the Massachusetts Eye and Ear Infirmary and the Noguchi Memorial Institute of Medical Research of the College of Health Sciences, University of Ghana.

POAG probands were unrelated and met the following three inclusion criteria: (1) intraocular pressure greater than 22 mm Hg in both eyes without medications or greater than 19 mm Hg with two or more medications; (2) glaucomatous optic neuropathy in both eyes; and (3) visual field loss consistent with optic nerve damage in at least one eye. Other affected members met at least 2 of these criteria. Glaucomatous optic neuropathy was defined as cup-to-disc ratio higher than 0.7 or focal loss of the nerve fiber layer (notch). Visual fields were performed by using standard automated perimetry. Exclusion criteria included the presence of any secondary form of glaucoma. Consenting family members who did not meet the above criteria were enrolled, but their genotypes were used only to establish linkage phase. The MYOC gene was sequenced in all probands. Families with disease-associated MYOC mutations were excluded from analysis. Age at diagnosis (AAD) was self-reported by POAG cases as the age at which they were first told by an eye specialist that they 1) had elevated intraocular pressure, 2) were prescribed IOP-lowering eye medications, or 3) had glaucoma.

### Sample preparation and genotyping

To conduct the genome screen, samples were genotyped using the Illumina GoldenGate Linkage 4 Panel or the Illumina Infinium Human Linkage-12 Panel. DNA from two CEPH individuals and two quality control samples were included in each 96-well plate used for genotyping, and a 100% match of these samples was required for inclusion of a marker in the analysis. A minimum genotyping efficiency of 95% was required for each marker.

Pedigree relationships were tested with RELPAIR [24], which uses identity-by-descent allele sharing estimates for statistical inference of biological relationships within and across the specified family structures. Discrepancies between specified and inferred relationships, typically due to sample switches, were addressed by removing four unresolved individuals and two families from the analysis. Genotypes that were inconsistent with Mendelian inheritance were identified with the program PEDCHECK [25] and removed prior to the linkage analysis.

### Whole genome linkage analysis

The final linkage analysis included 5233 single nucleotide polymorphisms (SNPs). Marker order and intermarker distances (in cM) were derived from the Decode linkage maps [26]. For markers not included in that panel, genetic distances were interpolated based on physical distances (1 cM∼1 Mb). The software MERLIN [27] was used to calculate nonparametric two-point and multipoint lod scores, using the exponential model and Spairs allele sharing statistic [28]. Parametric affecteds-only heterogeneity lod scores (HLODs) assuming a dominant (disease allele frequency 0.01) or recessive (disease allele frequency 0.2) model were also computed with MERLIN. For the separate analysis of Caucasian and African ancestry datasets, ethnicity-specific marker allele frequencies were estimated from all genotyped individuals [29]. To avoid an inflation of lod scores due to misspecified allele frequencies, particularly for markers with rare minor alleles in one of the two ethnicities, we also used these ethnicity-specific marker allele frequencies in the overall analysis. This was done by modifying the analysis files to include two “dummy markers” at the same map position as the real marker. The dummy markers had ethnicity-specific allele frequencies, with observed genotypes for samples of one ethnicity, but missing genotypes for the other. This computational approach allowed for the appropriate calculation of joint multipoint lod scores, which combine linkage information across map positions (i.e., at and in-between genotyped markers); joint two-point lod scores could not be calculated. To avoid an inflation of linkage evidence due to inter-marker LD in the absence of parental genotypes, we estimated haplotype frequencies of SNP clusters in high pairwise LD, using a threshold of r^2^ = 0.16 to define these clusters [30], [31].

### Ordered subset analysis (OSA)

Based on previous POAG linkage analyses [3], [22], we used OSA [21] to test whether AAD was a significant source of linkage heterogeneity in our dataset. Families were sorted by increasing average AAD in affected relatives and nonparametric linkage analysis was performed one family at a time in the AAD-based order until the family subset generating the maximum lod score anywhere on the given chromosome was identified. This maximum could occur at different map positions for different family subsets. Permutation testing was employed to test whether the observed increase in linkage evidence in this family subset was greater than expected by chance. To this end, families were randomly ordered and the lod score maximization procedure was repeated 10,000 times to calculate the proportion of random permutations with a maximum subset-based lod score at least as large as the observed one (empirical p-value). The OSA null hypothesis specifies no relationship between family-specific covariate and family-specific linkage evidence. Rejection of this null hypothesis suggests that the covariate, here AAD, is a statistically significant (empirical p-value≤0.05) source of linkage heterogeneity.
